# Knowledge and Needs of Resident Physicians Regarding Osteoporosis: A Nationwide Survey of Residents

**DOI:** 10.1002/jbm4.10524

**Published:** 2021-07-01

**Authors:** Carolyn J Crandall, Lucia Y Chen, Tyler D Rodriguez, David Elashoff, Stephanie S Faubion, Juliana M Kling, Jan Shifren, Lisa Skinner, Douglas C Bauer

**Affiliations:** ^1^ Department of Internal Medicine David Geffen School of Medicine at University of California, Los Angeles Los Angeles CA USA; ^2^ Department of Internal Medicine Mayo Clinic Jacksonville FL USA; ^3^ Department of Internal Medicine Mayo Clinic Scottsdale AZ USA; ^4^ Department of Obstetrics and Gynecology Massachusetts General Hospital, Harvard Medical School Boston MA USA; ^5^ Department of Internal Medicine University of California, San Francisco San Francisco CA USA

**Keywords:** FRACTURE, MEDICAL EDUCATION, OSTEOPOROSIS, RESIDENCY

## Abstract

Large‐scale studies have not addressed the knowledge level of US resident physicians regarding osteoporosis management. We gauged the knowledge level of family medicine, internal medicine, and obstetrics and gynecology resident physicians regarding osteoporosis management. In 2019, we sent an anonymous survey via e‐mail to all program directors of Accreditation Council for Graduate Medical Education–accredited residency programs in family medicine, internal medicine, and obstetrics and gynecology for distribution to resident physicians. Knowledge items assessed osteoporosis screening, diagnosis, and treatment. We received responses from 182 family medicine, 275 internal medicine, and 122 obstetrics and gynecology programs. Of 582 resident physician respondents, 31% were family medicine residents, 47% were internal medicine residents, and 21% were obstetrics and gynecology residents. Although 77% of respondents correctly selected the *T*‐score threshold for the diagnosis of osteoporosis among persons aged 50 years and older (−2.5), only 20% of respondents correctly identified minimal‐trauma hip fracture as being diagnostic of osteoporosis. One‐third of respondents correctly identified which medications were demonstrated in clinical trials to decrease hip fracture risk. Fifteen percent of respondents correctly identified that denosumab and alendronate are associated with osteonecrosis of the jaw; and 40% of respondents correctly identified that decline in bone density is more rapid after discontinuation of denosumab than after discontinuation of bisphosphonates. Less than half of resident physicians knew that bisphosphonate‐associated atypical femoral fractures are duration‐dependent. One‐quarter of respondents felt not at all prepared to manage osteoporosis. In this nationwide survey of resident physicians, knowledge regarding osteoporosis diagnosis and treatment was poor, with a striking lack of knowledge regarding the two most serious adverse effects of osteoporosis pharmacotherapy (osteonecrosis of the jaw and atypical femoral fractures). The undertreatment of osteoporosis is unlikely to improve without increased education of resident physicians. © 2021 The Authors. *JBMR Plus* published by Wiley Periodicals LLC on behalf of American Society for Bone and Mineral Research.

## 1 Introduction

One in two older women and one in three older men will experience osteoporosis‐related fractures in their remaining lifetimes.^(^ ^1^ ^)^ Given the complications associated with fractures, including limited ambulation, chronic pain and disability, loss of independence, and decreased quality of life,^(^ ^2^ ^)^ it is important to identify at‐risk individuals before the occurrence of fracture using evidence‐based screening and treatment guidelines. Rigorous evidence‐based guidelines are available regarding screening and treatment of osteoporosis. However, despite evidence‐based guidelines and available medical therapies, there is serious undertreatment of osteoporosis in the US. For example, treatment rates within 6 months after hip fracture have decreased from 15% in 2004 to 3% in 2015.^(^ ^3^ ^)^

The National Institutes of Health Pathways to Prevention Workshop in 2019 identified a critical need for more research on barriers to osteoporosis drug therapy, including who initiates drug treatment, who does not, and why, in addition to examining patient and provider attitudes regarding osteoporotic drug therapy.^(^ ^4^ ^)^ However, we hypothesize that one of the reasons for the “osteoporosis treatment gap” is insufficient exposure of resident physicians in primary care specialties to education regarding osteoporosis management. To our knowledge, results of a nationwide resident survey regarding knowledge and attitudes about osteoporosis screening and treatment have not been previously published.

The goals of this study were to (i) assess resident physicians' knowledge and competency in osteoporosis management, (ii) compare exposure to education regarding, and comfort level with, osteoporosis training among resident physicians of primary care specialties (internal medicine, family medicine, obstetrics and gynecology), and (iii) identify knowledge gaps in trainee curricula to highlight opportunities for improvement in the education of resident physicians. We hypothesized that certain characteristics would predict higher knowledge level and comfort with osteoporosis: self‐identification as female, internal medicine specialty, and higher postgraduate year (PGY) level.

## 2 Methods

### 2.1 Survey distribution

Between December 6, 2019, and March 12, 2020, we sent e‐mails to program directors of all of the Accreditation Council for Graduate Medical Education (ACGME)–accredited residency programs (282 obstetrics and gynecology programs, 675 family medicine programs, and 544 internal medicine programs) that were listed on the ACGME public‐access webpage on August 7, 2019. Programs that were newly accredited (had not yet enrolled resident physicians) were ineligible, leaving 250 obstetrics and gynecology, 528 family medicine, and 440 internal medicine programs eligible for survey. In the e‐mail, we requested the program directors of each program to distribute the uniform resource locator link to the survey and the accompanying cover letter explaining the anonymous voluntary web‐based survey to all resident physicians in their program on our behalf. In addition to sending the survey invitation to all the program e‐mail addresses listed on the ACGME website, we sought additional e‐mail addresses for the program directors using Google searches and PubMed searches. Up to two reminder e‐mails were sent to program directors from which no survey respondents reported being enrolled at that particular training program. The University of California, Los Angeles (UCLA) Redcap survey system was used to collect anonymous survey responses. Therefore, the research team did not have access to any identifiable data (such as names or e‐mail addresses of individual residents or individual survey responses). The UCLA Institutional Review Board designated this project as exempt from human subjects review.

### 2.2 Survey content

The anonymous web‐based survey was similar in its overall structure, length, and type of questions to that of another resident survey related to menopause management^(^ ^5^ ^)^ but with content based on current evidence‐based osteoporosis guidelines rather than menopause management guidelines (Supplemental Table S1). The anonymous survey included information regarding the goal of the study, 14 multiple‐choice knowledge questions based on content from the United States Preventive Services Task Force (USPSTF) osteoporosis screening guidelines^(^ ^1^, ^2^ ^)^ and the American College of Physicians (ACP) osteoporosis treatment guidelines,^(6^ ^)^ as well as questions regarding their age, gender identity, current PGY, medical specialty, number of patients with osteoporosis they manage in their continuity clinics, number of teaching sessions regarding osteoporosis during their training, source of education (specialty of preceptor providing the education), form of education (online, lecture, chalk talks, case‐based etc.), and level of comfort managing osteoporosis.

### 2.3 Statistical analysis

We categorized the results of the survey according to (i) specialty (internal medicine, family medicine, and obstetrics and gynecology); (ii) gender (female, male, other); and (iii) PGY level (≥3 versus <3). Six survey respondents listed “other” instead of internal medicine, obstetrics and gynecology, or family medicine; the stated specialties of these 6 respondents in the free‐response field were preliminary ophthalmology, traditional rotating internship, preliminary radiology, preliminary internship, medicine/pediatrics residency, and internal medicine/psychiatry. Data from those 6 respondents were excluded from the results that were stratified by program specialty. Missing responses to survey questions were considered as incorrect. We used chi‐square tests to compare frequencies of responses to knowledge questions by gender (female versus male) and PGY year (PGY <3 versus PGY ≥3). The frequency of “other” gender was too low (6 respondents) to allow reliable statistical analysis for this gender group, so these individuals were excluded from gender‐specific analyses. We used Fisher's exact tests to compare the proportions of responses by resident specialty (family medicine, internal medicine, and obstetrics and gynecology). We were unable to identify which internal medicine programs had a specific emphasis on primary care training.

## 3 Results

We received at least one response from 182 of 528 (34%) family medicine residency programs, 275 of 440 (63%) internal medicine residency programs, and 122 of 250 (49%) obstetrics and gynecology residency programs. We received at least one response from 48% (579 of 1218) of the residency programs. For academic year 2019–2020, there were an estimated 48,645 resident physicians in the specialties that we surveyed,^(^ ^7^ ^)^ corresponding to a response rate of 1% in terms of the proportion of resident respondents. Sixty‐two percent of the 582 respondents reported being female, 38% reported being male, and 1% self‐identified as other gender (Table 1). {TBL 1} Eight‐nine percent of respondents reported being between 26 and 35 years old. Thirty‐one percent reported being in a family medicine program, 47% were in an internal medicine program, and 21% were in an obstetrics and gynecology program. Sixty‐eight percent of respondents were in PGY year ≥3 and 32% of participants were in PGY year <3.

**Fig 1:**
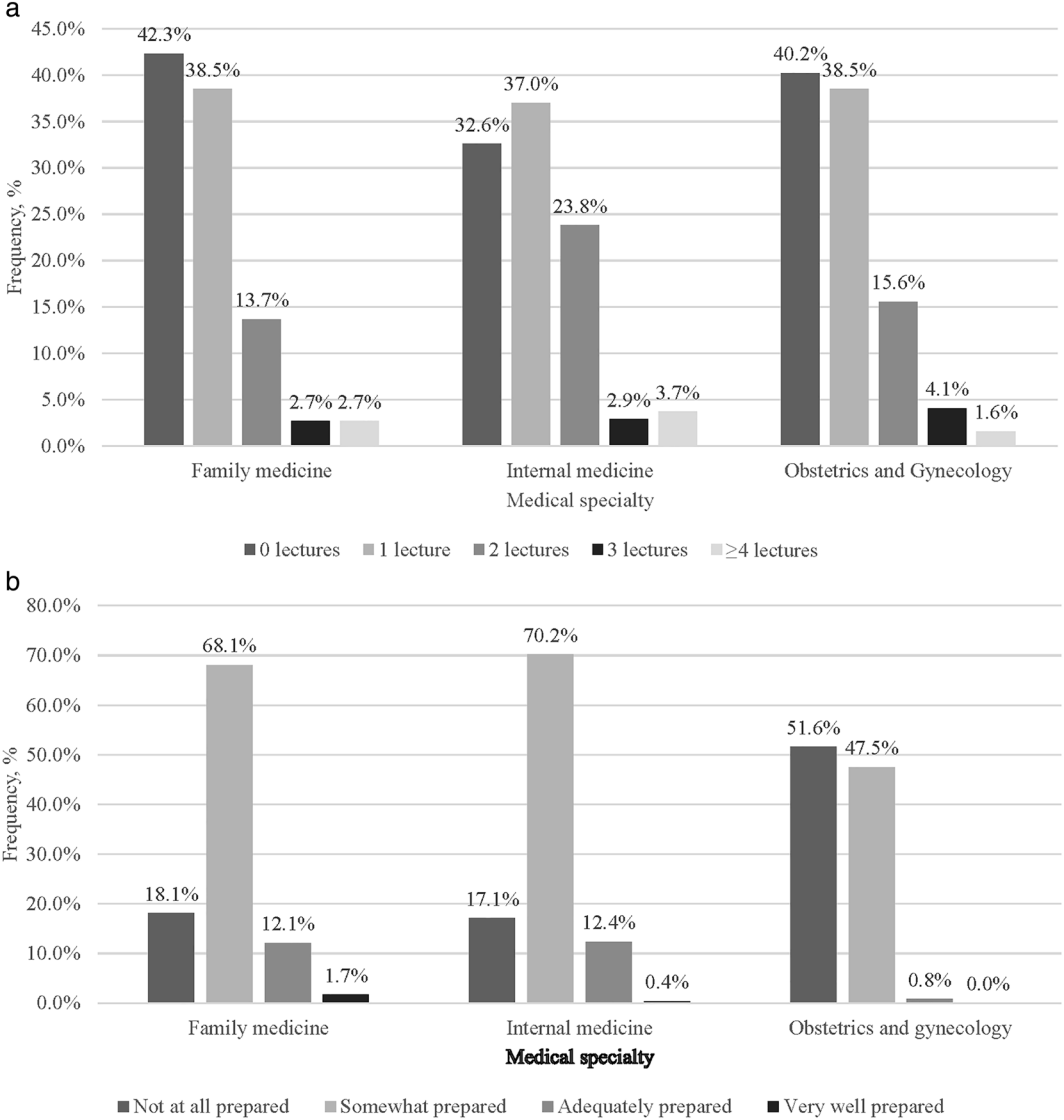
(*a*) Number of lectures or didactic sessions regarding osteoporosis management received during residency to date, as reported by residents. (*b*) Self‐reported degree of preparedness of resident physicians regarding osteoporosis.

**Table 1:** Gender, Specialty, and Postgraduate Year (PGY) of Survey Respondents

	% (*n*)
Gender	
Female	61.5% (358)
Male	37.5% (218)
Other	1.0% (6)
Total	100% (582)
Medical specialty	
Family medicine	31.1% (182)
Internal medicine	47.0% (275)
OB/GYN	20.9% (122)
Other	1.0% (6)
Total	100% (585)
PGY	
<3	68.0% (393)
≥3	32.0% (185)
Total	100% (578)
Age, overall (years)	
21 to 25	1.6% (9)
26 to 30	64.1% (370)
31 to 35	25.1% (145)
36 to 40	4.9% (28)
41 to 45	1.9% (11)
46 to 50	1.0% (6)
51 or older	1.4% (8)
Total	100% (577)

### 3.1 Knowledge regarding osteoporosis overall and by self‐reported gender

With regard to osteoporosis screening and diagnosis, 77% of respondents correctly believed that a *T*‐score ≤2.5 is the diagnostic criterion for osteoporosis among persons aged 50 years and older (Table 2). {TBL 2} A lower proportion of male (70%) than female (81%) respondents correctly identified the diagnostic bone mineral density (BMD) threshold (*p* = 0.004). Sixteen percent of respondents correctly identified the prevalence of osteoporosis‐related fractures in older women, with higher proportions of correct response in male (20%) than female (13%) respondents (*p* = 0.04). Only 10% of respondents correctly responded that mortality after hip fracture is higher in men than in women. One in 5 respondents selected the correct response regarding the proportion of hip fracture patients who regain pre‐fracture level of independence. Only 20% of respondents correctly identified that a 65‐year‐old otherwise healthy man with a minimal‐trauma hip fracture and normal BMD should be diagnosed with osteoporosis. The majority of residents knew that the USPSTF recommends beginning routine bone density screening at age 65 in women; more female than male resident physicians correctly answered this question (85% versus 73%, *p* < 0.001).

**Table 2:** Responses to Knowledge Questions by Gender

Item no.	Topic	Response^a^	Prevalence of selecting response (*n* [%])			*p* Value^b^
			Overall sample (*n* = 576)	Female (*n* = 358)	Male (*n* = 218)	
1	*T*‐score threshold for diagnosis of osteoporosis	** *T*‐score ≤ −2.5**	442 (77%)	289 (81%)	153 (70%)	0.004
		*T*‐score < −1	8 (1%)	4 (1%)	4 (2%)	
		*Z*‐score < −1	1 (0%)	0 (0%)	1 (0%)	
		*Z*‐score < −2.5	124 (22%)	64 (18%)	60 (28%)	
		Missing	1 (0%)	1 (0%)	0 (0%)	
2	Prevalence of osteoporosis‐related fractures in older women	10%	127 (22%)	84 (23%)	43 (20%)	
		20%	356 (62%)	224 (63%)	132 (61%)	
		**50%**	91 (16%)	48 (13%)	43 (20%)	0.04
		Missing	2 (0%)	2 (1%)	0 (0%)	
3	Mortality after hip fracture	Greater in women than in men	372 (65%)	232 (65%)	140 (64%)	
		**Greater in men than in women**	58 (10%)	30 (8%)	28 (13%)	0.08
		Equal in men and women	145 (25%)	95 (27%)	50 (23%)	
		Missing	1 (0%)	1 (0%)	0 (0%)	
4	Proportion of hip fracture patients who regain pre‐fracture level of independence	5%	185 (32%)	119 (33%)	66 (30%)	
		20%	273 (47%)	165 (46%)	108 (50%)	
		**40%**	117 (20%)	73 (20%)	44 (20%)	0.95
		Missing	1 (0%)	1 (0%)	0 (0%)	
5	Age to begin routine bone density screening in women (United States Preventive Services Task Force)	When they begin their menopausal transition	10 (2%)	5 (1%)	5 (2%)	
		One year after the menopausal transition	33 (6%)	13 (4%)	20 (9%)	
		**At age 65 years**	464 (81%)	304 (85%)	160 (73%)	<0.001
		At age 50 years	66 (11%)	34 (9%)	32 (15%)	
		Missing	3 (1%)	2 (1%)	1 (0%)	
6	Osteoporosis screening in men (United States Preventive Services Task Force)	Screen beginning at age 50 years	10 (2%)	4 (1%)	6 (3%)	
		Screen beginning at age 70 years	75 (13%)	48 (13%)	27 (12%)	
		**Insufficient evidence to recommend for or against screening**	430 (75%)	263 (73%)	167 (77%)	0.4
		None of the above	60 (10%)	42 (12%)	18 (8%)	
		1 (0%)	1 (0%)	0 (0%)		
7	Minimal‐trauma hip fracture is diagnostic of osteoporosis	**Osteoporosis**	115 (20%)	65 (18%)	50 (23%)	0.16
		Osteopenia	39 (7%)	24 (7%)	15 (7%)	
		Unclear, requires further studies	421 (73%)	268 (75%)	153 (70%)	
		Missing	1 (0%)	1 (0%)	0 (0%)	
8	Osteoporosis medications demonstrated in clinical trials to decrease hip fracture risk	Calcitonin	30 (5%)	20 (6%)	10 (5%)	
		Raloxifene	228 (40%)	169 (47%)	59 (27%)	
		Abaloparatide	109 (19%)	54 (15%)	55 (25%)	
		**None of the above**	208 (36%)	114 (32%)	94 (43%)	0.006
		Missing	1 (0%)	1 (0%)	0 (0%)	
9	First‐line therapy for osteoporosis (American College of Physicians guidelines)	Raloxifene	137 (24%)	94 (26%)	43 (20%)	
		Estrogen therapy	29 (5%)	16 (4%)	13 (6%)	
		Calcitonin	25 (4%)	19 (5%)	6 (3%)	
		**None of the above**	384 (67%)	228 (64%)	156 (72%)	0.05
		Missing	1 (0%)	1 (0%)	0 (0%)	
10	Optimal duration of osteoporosis pharmacotherapy	**Optimal duration of treatment is unclear**	365 (63%)	221 (62%)	144 (66%)	0.3
		Treatment duration is usually 10 years	91 (16%)	59 (16%)	32 (15%)	
		Adverse effects are not duration‐dependent	40 (7%)	23 (6%)	17 (8%)	
		None of the above	78 (14%)	54 (15%)	24 (11%)	
		Missing	2 (0%)	1 (0%)	1 (0%)	
11	Drug holiday definition	A switch from one osteoporosis medication to another	9 (2%)	3 (1%)	6 (3%)	
		**A temporary discontinuation of pharmacotherapy**	444 (77%)	279 (78%)	165 (76%)	0.53
		All of the above	122 (21%)	75 (21%)	47 (22%)	
		Missing	1 (0%)	1 (0%)	0 (0%)	
12	Most rapid bone density decline after discontinuation	**Denosumab**	232 (40%)	131 (37%)	101 (46%)	0.02
		Risedronate	78 (14%)	47 (13%)	31 (14%)	
		Alendronate	263 (46%)	178 (50%)	85 (39%)	
		Missing	3 (1%)	2 (1%)	1 (0%)	
13	Medications associated with osteonecrosis of the jaw	Denosumab	105 (18%)	64 (18%)	41 (19%)	
		Alendronate	382 (66%)	244 (68%)	138 (63%)	
		**All of the above**	88 (15%)	49 (14%)	39 (18%)	0.17
		Missing	1 (0%)	1 (0%)	0 (0%)	
14	Characteristics of bisphosphonate‐associated atypical femoral fractures	They are associated with use of raloxifene	48 (8%)	29 (8%)	19 (9%)	
		**They are duration‐dependent**	242 (42%)	159 (44%)	83 (38%)	0.13
		They occur in 10% of patients	153 (27%)	92 (26%)	61 (28%)	
		None of the above	128 (22%)	76 (21%)	52 (24%)	
		Missing	5 (1%)	2 (1%)	3 (1%)	

^a^ Boldface indicates correct responses.

^b^ The *p* values are calculated using chi‐square tests for comparison of correct answers between males and females.

With regard to osteoporosis therapy, 36% of respondents correctly identified that raloxifene, calcitonin, and abaloparatide have not been documented in randomized clinical trials to decrease hip fracture risk; a higher proportion of male (43%) than female (32%) respondents selected the correct response (*p* < 0.006). Two‐thirds of respondents correctly believed that raloxifene, estrogen therapy, and calcitonin are not first‐line therapies for osteoporosis according to ACP osteoporosis treatment guidelines. Only 40% of respondents correctly identified that BMD declines more rapidly after denosumab discontinuation than after bisphosphonate discontinuation, with a higher proportion of male (46%) than female (37%) respondents correctly answering this survey question (*p* = 0.02). Fifteen percent of respondents correctly identified that both denosumab use and alendronate use are associated with osteonecrosis of the jaw. Forty‐two percent of respondents identified that bisphosphonate‐associated atypical femoral fractures are duration‐dependent. (Twenty‐two percent of respondents incorrectly believed that atypical subtrochanteric and diaphyseal femoral fractures occur in 10% of individuals taking bisphosphonates.)

### 3.2 Knowledge regarding osteoporosis by PGY and medical specialty

Responses to osteoporosis knowledge‐related questions were generally similar among resident physicians in PGY ≥3 and those in PGY <3 (Table 3). {TBL 3} A higher proportion of PGY ≥3 respondents (29%) than PGY <3 respondents (16%) correctly identified that a 65‐year‐old otherwise healthy men with a minimal‐trauma hip fracture and normal BMD should be diagnosed with osteoporosis (*p* < 0.001). A higher proportion of PGY ≥3 respondents (73%) than PGY <3 respondents (64%) correctly identified that raloxifene, estrogen, and calcitonin are not first‐line therapies for osteoporosis according to ACP guidelines.

**Table 3:** Responses to Knowledge Questions by Postgraduate Year (PGY)

Item no.	Topic	Response^a^	Prevalence of selecting response (*n* [%])			
			Overall sample (*n* = 578)	PGY <3 (*n* = 393)	PGY ≥3 (*n* = 185)	*p* Value^b^
1	*T*‐score threshold for diagnosis of osteoporosis	** *T*‐score ≤ −2.5**	444 (77%)	302 (77%)	142 (77%)	0.98
		*T*‐score < −1	8 (1%)	6 (2%)	2 (1%)	
		*Z*‐score < −1	1 (0%)	0 (0%)	1 (1%)	
		*Z*‐score < −2.5	124 (21%)	84 (21%)	40 (22%)	
		Missing	1 (0%)	1 (0%)	0 (0%)	
2	Prevalence of osteoporosis‐related fractures in older women	10%	128 (22%)	80 (20%)	48 (26%)	
		20%	358 (62%)	251 (64%)	107 (58%)	
		**50%**	90 (16%)	60 (15%)	30 (16%)	0.77
		Missing	2 (0%)	2 (1%)	0 (0%)	
3	Mortality after hip fracture	Greater in women than in men	372 (64%)	248 (63%)	124 (67%)	
		**Greater in men than in women**	57 (10%)	37 (9%)	20 (11%)	0.6
		Equal in men and women	148 (26%)	107 (27%)	41 (22%)	
		Missing	1 (0%)	1 (0%)	0 (0%)	
4	Proportion of hip fracture patients who regain pre‐fracture level of independence	5%	185 (32%)	121 (31%)	64 (35%)	
		20%	273 (47%)	186 (47%)	87 (47%)	
		**40%**	119 (21%)	85 (22%)	34 (18%)	0.37
		Missing	1 (0%)	1 (0%)	0 (0%)	
5	Age to begin routine bone density screening in women (United States Preventive Services Task Force)	When they begin their menopausal transition	9 (2%)	8 (2%)	1 (1%)	
		One year after the menopausal transition	33 (6%)	22 (6%)	11 (6%)	
		**At age 65 years**	468 (81%)	311 (79%)	157 (85%)	0.1
		At age 50 years	65 (11%)	50 (13%)	15 (8%)	
		Missing	3 (1%)	2 (1%)	1 (1%)	
6	Osteoporosis screening in men (United States Preventive Services Task Force)	Screen beginning at age 50 years	10 (2%)	6 (2%)	4 (2%)	
		Screen beginning at age 70 years	77 (13%)	52 (13%)	25 (14%)	
		**Insufficient evidence to recommend for or against screening**	430 (74%)	292 (74%)	138 (75%)	0.94
		None of the above	60 (10%)	42 (11%)	18 (10%)	
		Missing	1 (0%)	1 (0%)	0 (0%)	
7	Minimal‐trauma hip fracture is diagnostic of osteoporosis	**Osteoporosis**	116 (20%)	62 (16%)	54 (29%)	<0.001
		Osteopenia	39 (7%)	30 (8%)	9 (5%)	
		Unclear, requires further studies	422 (73%)	300 (76%)	122 (66%)	
		Missing	1 (0%)	1 (0%)	0 (0%)	
8	Osteoporosis medications demonstrated in clinical trials to decrease hip fracture risk	Calcitonin	30 (5%)	25 (6%)	5 (3%)	
		Raloxifene	229 (40%)	152 (39%)	77 (42%)	
		Abaloparatide	109 (19%)	69 (18%)	40 (22%)	
		**None of the above**	209 (36%)	146 (37%)	63 (34%)	0.47
		Missing	1 (0%)	1 (0%)	0 (0%)	
9	First‐line therapy for osteoporosis (American College of Physicians guidelines)	Raloxifene	139 (24%)	103 (26%)	36 (19%)	
		Estrogen therapy	27 (5%)	20 (5%)	7 (4%)	
		Calcitonin	25 (4%)	18 (5%)	7 (4%)	
		**None of the above**	386 (67%)	251 (64%)	135 (73%)	0.03
		Missing	1 (0%)	1 (0%)	0 (0%)	
10	Optimal duration of osteoporosis pharmacotherapy	**Optimal duration of treatment is unclear**	364 (63%)	239 (61%)	125 (68%)	0.12
		Treatment duration is usually 10 years	93 (16%)	70 (18%)	23 (12%)	
		Adverse effects are not duration‐dependent	41 (7%)	30 (8%)	11 (6%)	
		None of the above	78 (13%)	52 (13%)	26 (14%)	
		Missing	2 (0%)	2 (1%)	0 (0%)	
11	Drug holiday definition	A switch from one osteoporosis medication to another	9 (2%)	7 (2%)	2 (1%)	
		**A temporary discontinuation of pharmacotherapy**	445 (77%)	307 (78%)	138 (75%)	0.35
		All of the above	123 (21%)	78 (20%)	45 (24%)	
		Missing	1 (0%)	1 (0%)	0 (0%)	
12	Most rapid bone density decline after discontinuation	**Denosumab**	231 (40%)	164 (42%)	67 (36%)	0.21
		Risedronate	78 (13%)	54 (14%)	24 (13%)	
		Alendronate	264 (46%)	170 (43%)	94 (51%)	
		Missing	5 (1%)	5 (1%)	0 (0%)	
13	Medications associated with osteonecrosis of the jaw	Denosumab	107 (19%)	80 (20%)	27 (15%)	
		Alendronate	383 (66%)	260 (66%)	123 (66%)	
		**All of the above**	87 (15%)	52 (13%)	35 (19%)	0.07
		Missing	1 (0%)	1 (0%)	0 (0%)	
14	Characteristics of bisphosphonate‐associated atypical femoral fractures	They are associated with use of raloxifene	47 (8%)	32 (8%)	15 (8%)	
		**They are duration‐dependent**	242 (42%)	171 (44%)	71 (38%)	0.24
		They occur in 10% of patients	157 (27%)	108 (27%)	49 (26%)	
		None of the above	127 (22%)	78 (20%)	49 (26%)	
		Missing	5 (1%)	4 (1%)	1 (1%)	

^a^ Boldface indicates correct responses.

^b^ The *p* values are calculated using chi‐square tests for comparison of correct answers between PGY <3 and PGY ≥3 residents.

Responses to several of the osteoporosis knowledge‐related questions differed by medical specialty (Table 4). {TBL 4} Regarding osteoporosis epidemiology and screening, a lower proportion of obstetrics and gynecology residents (5%) than family medicine (15%) or internal medicine (20%) residents knew the prevalence of osteoporosis‐related fractures in older women (*p* = 0.006 for obstetrics and gynecology versus family medicine; *p* < 0.001 for obstetrics and gynecology versus internal medicine). A lower proportion of obstetrics and gynecology (69%) than internal medicine (71%) or family medicine residents (84%) knew the USPSTF guidelines recommendation about osteoporosis screening in men (*p* = 0.003 for obstetrics and gynecology versus family medicine; *p* = 0.68 for obstetrics and gynecology versus internal medicine). Conversely, a higher proportion of obstetrics and gynecology (94%) than internal medicine (75%) or family medicine residents (81%) knew the age at which routine osteoporosis screening should begin according to USPSTF guidelines (*p* = 0.001 for obstetrics and gynecology versus family medicine; *p* < 0.001 for obstetrics and gynecology versus internal medicine).

**Table 4:** Responses to Knowledge Questions by Medical Specialty

Item no.	Topic	Response^a^	Prevalence of selecting response (*n* [%])				*p* Value^b^
			Overall sample (*n* = 579)	Family medicine (*n* = 182; 31.4%)	Internal medicine (*n* = 275; 47.5%)	OB/GYN (*n* = 122; 21.1%)	
1	*T*‐score threshold for diagnosis of osteoporosis	** *T*‐score ≤ −2.5**	445 (77%)	137 (75%)	215 (78%)	93 (76%)	0.76
		*T*‐score < −1	8 (1%)	4 (2%)	1 (0%)	3 (2%)	
		*Z*‐score < −1	1 (0%)	0 (0%)	0 (0%)	1 (1%)	
		*Z*‐score < −2.5	124 (21%)	41 (23%)	59 (21%)	24 (20%)	
		Missing	1 (0%)	0 (0%)	0 (0%)	1 (1%)	
2	Prevalence of osteoporosis‐related fractures in older women	10%	127 (22%)	35 (19%)	47 (17%)	45 (37%)	
		20%	363 (63%)	120 (66%)	172 (63%)	71 (58%)	
		**50%**	87 (15%)	27 (15%)	54 (20%)	6 (5%)	<0.001
		Missing	2 (0%)	0 (0%)	2 (1%)	0 (0%)	
3	Mortality after hip fracture	Greater in women than in men	374 (65%)	114 (63%)	159 (58%)	101 (83%)	
		**Greater in men than in women**	58 (10%)	17 (9%)	34 (12%)	7 (6%)	0.12
		Equal in men and women	146 (25%)	51 (28%)	81 (29%)	14 (11%)	
		Missing	1 (0%)	0 (0%)	1 (0%)	0 (0%)	
4	Proportion of hip fracture patients who regain pre‐fracture level of independence	5%	184 (32%)	52 (29%)	92 (33%)	40 (33%)	
		20%	273 (47%)	89 (49%)	125 (45%)	59 (48%)	
		**40%**	121 (21%)	41 (23%)	57 (21%)	23 (19%)	0.74
		Missing	1 (0%)	0 (0%)	1 (0%)	0 (0%)	
5	Age to begin routine bone density screening in women (United States Preventive Services Task Force)	When they begin their menopausal transition	10 (2%)	6 (3%)	4 (1%)	0 (0%)	
		One year after the menopausal transition	32 (6%)	9 (5%)	22 (8%)	1 (1%)	
		**At age 65 years**	470 (81%)	148 (81%)	207 (75%)	115 (94%)	<0.001
		At age 50 years	66 (11%)	19 (10%)	41 (15%)	6 (5%)	
		Missing	1 (0%)	0 (0%)	1 (0%)	0 (0%)	
6	Osteoporosis screening in men (United States Preventive Services Task Force)	Screen beginning at age 50 years	11 (2%)	2 (1%)	7 (3%)	2 (2%)	
		Screen beginning at age 70 years	75 (13%)	12 (7%)	41 (15%)	22 (18%)	
		**Insufficient evidence to recommend for or against screening**	431 (74%)	152 (84%)	195 (71%)	84 (69%)	0.003
		None of the above	61 (11%)	16 (9%)	31 (11%)	14 (11%)	
		Missing	1 (0%)	0 (0%)	1 (0%)	0 (0%)	
7	Minimal‐trauma hip fracture is diagnostic of osteoporosis	**Osteoporosis**	116 (20%)	29 (16%)	66 (24%)	21 (17%)	0.07
		Osteopenia	39 (7%)	13 (7%)	18 (7%)	8 (7%)	
		Unclear, requires further studies	423 (73%)	140 (77%)	190 (69%)	93 (76%)	
		Missing	1 (0%)	0 (0%)	1 (0%)	0 (0%)	
8	Osteoporosis medications demonstrated in clinical trials to decrease hip fracture risk	Calcitonin	31 (5%)	15 (8%)	10 (4%)	6 (5%)	
		Raloxifene	226 (39%)	66 (36%)	83 (30%)	77 (63%)	
		Abaloparatide	108 (19%)	33 (18%)	57 (21%)	18 (15%)	
		**None of the above**	213 (37%)	68 (37%)	124 (45%)	21 (17%)	<0.001
		Missing	1 (0%)	0 (0%)	1 (0%)	0 (0%)	
9	First‐line therapy for osteoporosis (American College of Physicians guidelines)	Raloxifene	138 (24%)	47 (26%)	40 (15%)	51 (42%)	
		Estrogen therapy	29 (5%)	5 (3%)	17 (6%)	7 (6%)	
		Calcitonin	26 (4%)	7 (4%)	12 (4%)	7 (6%)	
		**None of the above**	385 (66%)	123 (68%)	205 (75%)	57 (47%)	<0.001
		Missing	1 (0%)	0 (0%)	1 (0%)	0 (0%)	
10	Optimal duration of osteoporosis pharmacotherapy	**Optimal duration of treatment is unclear**	362 (63%)	118 (65%)	165 (60%)	79 (65%)	0.49
		Treatment duration is usually 10 years	96 (17%)	22 (12%)	52 (19%)	22 (18%)	
		Adverse effects are not duration‐dependent	41 (7%)	10 (5%)	25 (9%)	6 (5%)	
		None of the above	78 (13%)	32 (18%)	31 (11%)	15 (12%)	
		Missing	2 (0%)	0 (0%)	2 (1%)	0 (0%)	
11	Drug holiday definition	A switch from one osteoporosis medication to another	8 (1%)	1 (1%)	6 (2%)	1 (1%)	
		**A temporary discontinuation of pharmacotherapy**	443 (77%)	151 (83%)	199 (72%)	93 (76%)	0.03
		All of the above	127 (22%)	30 (16%)	69 (25%)	28 (23%)	
		Missing	1 (0%)	0 (0%)	1 (0%)	0 (0%)	
12	Most rapid bone density decline after discontinuation	**Denosumab**	229 (40%)	48 (26%)	152 (55%)	29 (24%)	<0.001
		Risedronate	79 (14%)	33 (18%)	29 (11%)	17 (14%)	
		Alendronate	266 (46%)	101 (55%)	90 (33%)	75 (61%)	
		Missing	5 (1%)	0 (0%)	4 (1%)	1 (1%)	
13	Medications associated with osteonecrosis of the jaw	Denosumab	106 (18%)	37 (20%)	45 (16%)	24 (20%)	
		Alendronate	386 (67%)	122 (67%)	178 (65%)	86 (70%)	
		**All of the above**	86 (15%)	23 (13%)	51 (19%)	12 (10%)	0.047
		Missing	1 (0%)	0 (0%)	1 (0%)	0 (0%)	
14	Characteristics of bisphosphonate‐associated atypical femoral fractures	They are associated with use of raloxifene	48 (8%)	15 (8%)	27 (10%)	6 (5%)	
		**They are duration‐dependent**	245 (42%)	75 (41%)	115 (42%)	55 (45%)	0.78
		They occur in 10% of patients	156 (27%)	53 (29%)	72 (26%)	31 (25%)	
		None of the above	125 (22%)	38 (21%)	57 (21%)	30 (25%)	
		Missing	5 (1%)	1 (1%)	4 (1%)	0 (0%)	

^a^ Boldface indicates correct responses.

^b^ The *p* values are calculated using chi‐square tests for comparison of correct answers between family medicine, internal medicine, and OB/GYN residents.

Regarding osteoporosis therapy, a lower proportion of obstetrics and gynecology residents (17%) than family medicine (37%) or internal medicine (45%) residents correctly identified which osteoporosis medications were demonstrated in clinical trials to decrease hip fracture risk (*p* < 0.001 for obstetrics and gynecology versus family medicine; *p* < 0.001 for obstetrics and gynecology versus internal medicine). A lower proportion of obstetrics and gynecology (47%) than family medicine (68%) or internal medicine (75%) residents correctly identified the first‐line therapies for osteoporosis according to ACP osteoporosis treatment guidelines (*p* < 0.001 for obstetrics and gynecology versus family medicine; *p* < 0.001 for obstetrics and gynecology versus internal medicine). A higher proportion of internal medicine residents (55%) than family medicine (26%) or obstetrics and gynecology (24%) residents correctly identified that BMD declines most rapidly after denosumab discontinuation than after bisphosphonate discontinuation (*p* < 0.001 for internal medicine versus family medicine; *p* < 0.001 for internal medicine versus obstetrics and gynecology).

### 3.3 Comfort level and sources of osteoporosis education reported by resident physicians

More than 40% of respondents reported receiving no lectures/didactics regarding osteoporosis management during residency training (Table 5, Figure 1). {TBL 5} Even among resident physicians in PGY ≥3, a quarter had not attended any didactic lectures regarding osteoporosis during their residency training. Numbers of lectures regarding osteoporosis were low across residency specialty. A higher proportion of internal medicine residents than obstetrics and gynecology or family medicine residents reported receiving two lectures about osteoporosis during residency (*p* = 0.07 for internal medicine versus obstetrics and gynecology; *p* = 0.009 for internal medicine versus family medicine). Overall, there was a pattern of respondents receiving most of their osteoporosis education from specialties corresponding to their residency specialty. Because osteoporosis is particularly common among postmenopausal women, the survey included an item about training in menopausal medicine. One‐fifth of respondents stated that menopause‐related medicine was not included in their curriculum to date, with a difference by specialty (internal medicine 27%, obstetrics and gynecology 14.0%, family medicine 18.9%, Fisher's exact test *p* = 0.001).

**Table 5:** Numbers of Patients With Osteoporosis, Comfort Level Regarding Managing Osteoporosis, Osteoporosis Education (Number of Lectures, Specialty of Preceptor, and Educational Format) During Training as Reported by Residents, by Respondent Specialty

	Total % (*n*)	Specialty of respondent^a^		
		Family medicine	Internal medicine	OB/GYN
No. of lectures attended regarding osteoporosis during residency (question 15)				
0	37.1% (215)	42.3% (77)	32.4% (89)	40.2% (49)
1	37.7% (218)	38.5% (70)	36.7% (101)	38.5% (47)
2	18.9% (109)	13.7% (25)	23.6% (65)	15.6% (19)
3	3.1% (18)	2.7% (5)	2.9% (8)	4.0% (5)
More than 4	2.9% (17)	2.7% (5)	3.6% (10)	1.6% (2)
Unspecified	0.3% (2)	0.0% (0)	0.7% (2)	0.0% (0)
Total	100% (579)	100% (182)	100% (275)	100% (122)
Medical specialty of preceptors providing most of osteoporosis education during residency (question 16)				
Endocrinology preceptor	12.3% (71)	2.2% (4)	23.4% (64)	2.5% (3)
Family medicine preceptor	25.7% (148)	74.2% (135)	3.7% (10)	2.5% (3)
General internal medicine preceptor	27.4% (158)	2.7% (5)	54.2% (148)	4.1% (5)
Obstetrics and gynecology preceptor	15.6% (90)	3.3% (6)	0.0% (0)	69.4% (84)
Rheumatology preceptor	4.7% (27)	2.2% (4)	8.4% (23)	0.0% (0)
Other	14.2% (82)	15.4% (28)	10.3% (28)	21.5% (26)
Total	100% (576)	100% (182)	100% (273)	100% (121)
Format of menopause‐related education during residency training^b^ (question 17)				
Online/modules	12.7% (103)	9.1% (24)	17.9% (64)	8.1% (15)
Lecture	36.7% (296)	39.3% (104)	29.3% (105)	46.8% (87)
Simulation center	0.2% (2)	0.0% (0)	0.3% (1)	4.1% (1)
Chalk talks	6.3% (51)	6.4% (17)	0.0% (20)	0.5% (14)
Case‐based teaching	20.1% (163)	22.7% (60)	5.6% (67)	19.4% (36)
It was not included	21.3% (172)	18.9% (50)	26.8% (96)	14.0% (26)
Other	2.6% (21)	3.4% (9)	1.4% (5)	3.7% (7)
Total	100% (808)	100% (264)	100% (358)	100% (186)
No. of patients with osteoporosis cared for in continuity clinic during residency (question 18)				
None	17.1% (99)	11.5% (21)	10.9% (30)	39.3% (48)
1–5	39.8% (230)	41.2% (75)	37.6% (103)	42.6% (52)
6–10	20.8% (120)	23.6% (43)	23.4% (64)	10.7% (13)
11–20	12.6% (73)	14.3% (26)	15.0% (41)	4.9% (6)
21–30	4.7% (27)	4.9% (9)	6.2% (17)	0.8% (1)
More than 30	5.0% (29)	4.4% (8)	6.9% (19)	1.6% (2)
Total	100% (578)	100% (182)	100% (274)	100% (122)
How prepared do you feel to manage patients with osteoporosis? (question 19)				
Not at all prepared	24.7%	18.1%	17.1%	51.6%
Total	579	182	275	122

^a^ Thirteen participants did not provide information regarding their medical specialty.

^b^ Respondents could select as many types of lecture media that applied to them.

One‐quarter of respondents felt not at all prepared to manage patients with osteoporosis. A significantly higher proportion of obstetrics and gynecology residents (52%) compared with family medicine (18%) and internal medicine (17%) physicians reported feeling not at all prepared to manage patients with osteoporosis (*p* < 0.01). A higher proportion (39%) of obstetrics and gynecology residents reporting not caring for any patients with osteoporosis in their continuity clinics compared with 12% in family medicine respondents and 11% in internal medicine respondents (*p* < 0.001).

## 4 Discussion

In this nationwide survey of resident physicians training in family medicine, internal medicine, and obstetrics and gynecology, knowledge regarding osteoporosis was poor across the spectrum of diagnosis and treatment. Particularly striking was the high frequency of the incorrect belief that a *Z*‐score ≤2.5 is the diagnostic criterion for the diagnosis of osteoporosis and the lack of knowledge regarding the two most serious adverse effects of osteoporosis pharmacotherapy: osteonecrosis of the jaw and atypical femoral fractures. We did not find many notable gender differences in osteoporosis knowledge, but we found several differences by medical specialty. To our knowledge, a nationwide survey of residents' knowledge regarding osteoporosis management in the US has not been previously published.

We also found differences in self‐assessed preparedness and knowledge regarding osteoporosis management across medical specialties. Although only 17% of internal medicine residents felt “not at all prepared to manage osteoporosis,” knowledge level was generally poor among internal medicine residents, suggesting a “mismatch” between knowledge level and clinical “comfort level” in osteoporosis management. Half of obstetrics and gynecology residents felt not at all prepared to manage patients with osteoporosis. Internal medicine and family medicine residents were more likely than obstetrics and gynecology residents to select correct answers in some survey items regarding the efficacy of osteoporosis therapy (particularly regarding which drugs reduce hip fracture risk), but knowledge was poor regardless of specialty. Even among PGY ≥3 resident physicians, osteoporosis‐related knowledge was suboptimal.

Although female residents scored better than male residents regarding BMD threshold for osteoporosis, the prevalence of incorrect responses by female resident physicians was high. Comparisons of responses of male versus female residents may be confounded by specialty area, as obstetrics and gynecology residents are predominantly female.

Given the knowledge gaps that we identified regarding osteoporosis, it was not surprising that one‐quarter of resident physicians felt not at all prepared to manage osteoporosis. These results suggest that resident physicians would be open to learning more about osteoporosis management if the education were provided.

To our knowledge, this study is the first published description of a nationwide US resident physician survey regarding osteoporosis knowledge. We were also unable to find published studies regarding osteoporosis knowledge levels among practicing physicians in the US. One survey of primary care physicians in the US assessed attitudes: Only 36% to 45% of physicians surveyed recommended treatment for patients whose history and BMD placed them below guideline‐recommended treatment threshold.^(^ ^8^ ^)^ Systematic reviews have not emphasized physician knowledge in relation to osteoporosis^(^ ^9^ ^)^ and have emphasized the impact of decision aids versus usual care on treatment of osteoporosis.^(^ ^10^ ^)^

The current results suggesting gaps in resident physician knowledge about osteoporosis management mirror findings of studies evaluating resident knowledge of management of other common conditions. For example, significant knowledge gaps regarding management of chronic kidney disease were identified in a survey of internal medicine, internal medicine/pediatrics, and family medicine residents.^(^ ^11^ ^)^ Similarly, internal medicine house‐staff had poor awareness and knowledge for dose adjustment for common cardiovascular drugs in patients with chronic kidney disease,^(^ ^12^ ^)^ and specific gaps in knowledge of chronic kidney disease guidelines.^(^ ^13^ ^)^ Finally, a study investigating knowledge about menopause management among all levels of internal medicine, family medicine, and obstetrics and gynecology residents showed that despite the belief that training in menopause management was important, knowledge gaps were present, and a minority felt adequately prepared to manage women experiencing menopause.^(^ ^5^ ^)^

In addition to highlighting knowledge gaps about osteoporosis management among resident physicians, these results will inform specific ways to modify postgraduate training to improve osteoporosis management. First, the number of didactic sessions regarding osteoporosis should be increased; in our study, more than one‐third of residents reported that they did not experience any didactic sessions on osteoporosis during residency training, and even among resident physicians in PGY ≥3, a quarter had not attended any didactic lectures regarding osteoporosis during their residency training. The scant education reported by resident physicians was typically provided by physicians in their corresponding specialty (obstetrics and gynecology preceptor for obstetrics and gynecology residents, family medicine preceptor for family medicine resident, general internal medicine preceptor for internal medicine residents). Improving knowledge of resident physicians regarding osteoporosis is necessary but not likely to be sufficient. We are unaware of comparative studies that have established the optimal method to improve knowledge in residents. Lectures, mentoring, hands‐on interdisciplinary learning, and standardized testing may all play a role. Future studies should compare educational venues and preceptors' medical specialties to identify the best and most time‐efficient method for resident physicians to learn about osteoporosis management.

Given the low proportion of individuals with hip fracture who receive therapy within 6 months of hospital discharge, it is likely that a survey of orthopedic residents similar to the one described in our current study would be valuable in documenting the needs and knowledge level of orthopedic resident physicians.

Our results have clinical implications. An estimated 12 million individuals in the US older than 50 years have osteoporosis.^(^ ^2^ ^)^ One in two older women and one in three older men will experience osteoporosis‐related fractures in their remaining lifetimes, and osteoporosis remains seriously undertreated, particularly in the secondary prevention setting. However, we found that the majority of resident physicians were unaware of the fact that minimal‐trauma hip fracture is diagnostic of osteoporosis; did not know which of the FDA‐approved medications have been demonstrated to reduce risk of hip fracture; were unaware of the rapid offset of BMD effects after denosumab discontinuation; and did not know that both denosumab use and alendronate use are associated with increased risk of osteonecrosis of the jaw and that bisphosphonate‐associated atypical femoral fractures are duration‐dependent. These knowledge gaps could have substantial clinical ramifications: Lack of timely identification of hip fracture patients for osteoporosis treatment, initiating osteoporosis medications that are not first‐line agents, and not counseling patients appropriately about duration‐dependent serious adverse effects of antiresorptive medications. If the resident physicians are unaware of rebound vertebral fractures after denosumab discontinuation and the need to replace denosumab with another antiresorptive (typically a bisphosphonate), there could be harm to patients. Closure of these gaps will require improving education regarding osteoporosis management across medical specialties.

Our study has several strengths. We invited program directors from every ACGME‐accredited family medicine, internal medicine, and obstetrics and gynecology training program in the US to distribute our survey, enhancing the representativeness of our results. We sent the invitation to the e‐mail addresses of all of the programs listed on the ACGME website and sought additional e‐mail addresses from other publicly available data sources. We included family medicine, internal medicine, and obstetrics and gynecology residents, capturing the medical specialties that would be expected to assume responsibility for osteoporosis management in the future. We sent reminders to program directors if we had not received any responses from residents of their program. We used an anonymous survey that we believed would be likely to elicit honest survey responses.

Potential limitations of our study include that because program directors distributed the survey to resident physicians, we could not contact individual residents to remind them to complete the anonymous survey if they have not already responded. Also, resident physicians have numerous commitments and time constraints; limited response rates are a challenge with all resident surveys. Because the survey was anonymous, we cannot rule out differences in osteoporosis knowledge among respondents versus non‐respondents either within or across institutions. However, we do not believe that the suboptimal response invalidates the primary results of our study because (i) non‐respondents were likely busier or less interested in the content of the survey and therefore unlikely to have sought additional training related to osteoporosis; and (ii) it is highly unlikely that non‐respondents have greater confidence in their ability of manage osteoporosis. Responses of practicing physicians generally show low response rates.^(^ ^14^ ^)^ We received at least one response from 48% (579 of 1218) of the residency programs, which would be considered a favorable response for survey studies. However, for academic year 2019–2020, there were an estimated 48,645 resident physicians in the specialties that we surveyed,^(^ ^7^ ^)^ corresponding to a response rate of 1% in terms of the proportion of resident respondents. Finally, we could not confirm the actual number of didactics and other sessions provided to resident physicians.

In conclusion, our results demonstrate poor knowledge about osteoporosis management among resident physicians in internal medicine, family medicine, and obstetrics and gynecology. Our findings suggest that one potential way to close the “osteoporosis treatment gap”^(^ ^15^, ^16^ ^)^ is to improve education of resident physicians in the specialties of family medicine, internal medicine, and obstetrics and gynecology, in whose purview osteoporosis diagnosis and management lies. Given the rapidly increasing aging population in the US, with corresponding exponential increases in hip fractures, this need will only grow.

## Disclosures

All authors state that they have no conflicts of interest.

## Supporting information


**Appendix** **S1.** Supporting information
