# Supplementary material for: Knowledge and Needs of Resident Physicians Regarding Osteoporosis: A Nationwide Survey of Residents
Source: JBMR Plus. 2021 Jul 1;5(8):e10524. doi: 10.1002/jbm4.10524 (PMC8328797; doi:10.1002/jbm4.10524)
Supplement: Supplementary file 1 — Appendix S1. Supporting information [file JBM4-5-e10524-s001.pdf]

Knowledge and Needs of Residents Regarding Osteoporosis: A Nationwide Survey of U.S. Residents in Internal Medicine, Gynecology, and Family Medicine

Carolyn J. Crandall, MD, MS, FACP, Tyler D. Rodriguez, David Elashoff, PhD, Stephanie Faubion, MD, MBA, Jewel Kling, MD, MPH, Jan Shifren, MD, Lisa Skinner, MD, Douglas Bauer, MD

Supplemental Table 1. Survey Questionnaire[a]

Osteoporosis Management Knowledge in Post-Graduate Residents

INTRODUCTION

The purpose of this research survey is to assess your training in, and comfort level with, the screening and management of osteoporosis. The survey should take less than 10 minutes to complete. You will not be graded. Your responses are completely confidential and de-identified. The survey results will be used only to make recommendations for improving residency curricula so that you are prepared to counsel patients regarding osteoporosis screening and treatment. Your participation is completely voluntary. You have the right to withdraw your consent of discontinue participation at any time without penalty. Your current and future employment will not be affected by whether or not you participate. We sincerely hope that you will take a few minutes to complete the survey.

If you have any questions or concerns, please free to contact me (ccrandall@mednet.ucla.edu). If you should encounter problems in filling out the survey itself, please contact Tyler at the REDCap Survey Center, his email is TDRodriguez@mednet.ucla.edu.

Thank you for your participation!

Knowledge and Competency

1. The diagnosis of osteoporosis in persons aged 50 year and older by dual-energy x-ray absorptiometry (DXA) requires which of the following?

   ☐**T-score less than or equal to -2.5**

   ☐T-score less than -1

   ☐Z-score less than -1

   ☐Z-score less than -2.5

2. The prevalence of osteoporosis-related fracture in older women is approximately?

   ☐10%

[a] Correct answers are in boldface type

☐20%

☐**50%**

3. Mortality after hip fracture is:

   ☐Greater in women than in men

   ☐**Greater in men than in women**

   ☐Equal in men and women

4. After hip fracture, what proportion of persons regain their pre-fracture level of independence:

   ☐5%

   ☐20%

   ☐**40%**

5. The United States Preventive Services Task Force recommends routine screening for osteoporosis with bone density measurement among women starting:

   ☐When they begin their menopausal transition

   ☐One year after the menopausal transition

   ☐**At age 65**

   ☐At age 50

6. The United States Preventive Services Task Force recommends the following for men:

   ☐Screen beginning at age 50

   ☐Screen beginning at age 70

   ☐**Insufficient evidence to recommend for or against screening**

   ☐None of the above

7. An otherwise healthy 65-year old man experiences a hip fracture after tripping over a loose wire. His DXA test reveals normal bone density. What is his diagnosis?

   ☐**Osteoporosis**

   ☐Osteopenia

   ☐Unclear, requires further studies

8. Which of the following medications is demonstrated in randomized trials to decrease hip fracture among persons with osteoporosis?

   ☐Calcitonin

   ☐Raloxifene

   ☐Abaloparatide

   ☐**None of the above**

9. According to the American College of Physicians osteoporosis treatment guidelines, which of the following medications is first-line therapy for osteoporosis in women?

   ☐Raloxifene

   ☐Estrogen therapy

   ☐Calcitonin

   ☐**None of the above**

10. Which of the following is true regarding osteoporosis pharmacotherapy?

    ☐**Optimal duration of treatment is unclear**

    ☐Treatment duration is usually 10 years

    ☐Adverse effects are not duration-dependent

    ☐None of the above

11. In the context of osteoporosis pharmacotherapy, a "drug holiday" is:

    ☐A switch from one osteoporosis medication to another

☐**A temporary discontinuation of pharmacotherapy**

☐All of the above

12. Bone density declines most rapidly after cessation of:

☐**Denosumab**

☐Risedronate

☐Alendronate

13. Osteonecrosis of the jaw (ONJ) is associated with which of the following medications:

☐Denosumab

☐Alendronate

☐**All of the above**

14. Which of the following is true regarding bisphosphonate-associated atypical subtrochanteric and diaphyseal femoral fractures?

☐They are associated with use of raloxifene

☐**They are duration-dependent**

☐They occur in 10% of patients

☐None of the above

15. How many lectures/didactics regarding osteoporosis management have you received during your residency training to date?

☐0

☐1

☐2

☐3

☐More than 4

16. During your residency training, which of the following is the source of most of your osteoporosis education?

☐Endocrinology preceptor

☐Rheumatology preceptor

☐General Internal Medicine preceptor

☐Obstetrics and Gynecology preceptor

☐Family Medicine preceptor

☐Other (please specify):

17. How have you seen concepts related to menopause medicine being integrated into your resident training? Choose all that apply.

☐Online/modules

☐Lecture

☐Simulation center

☐Chalk talks

☐Case-based teaching

☐It was not included

☐Other (please specify)

18. How many patients with osteoporosis have you cared for in your continuity clinic during residency?

☐ 1 to 5

☐ 6 to 10

☐ 11 to 20

☐ 21 to 30

☐ More than 30

☐ None

19. How prepared do you feel to manage patients with osteoporosis?

☐ Not at all prepared

☐ Somewhat prepared

☐ Adequately prepared

☐ Very well prepared

20. Which training program are you enrolled in?

☐ Internal medicine

☐ Family medicine

☐ Ob/GYN

☐ Other:

21. Which PGY year are you?

☐ PGY 1

☐ PGY 2

☐ PGY 3

☐ PGY 4

☐ PGY 5

☐ PGY 6

☐ Other:

22. What is your age?

☐ 21 to 25

☐ 26 to 30

☐ 31 to 35

☐ 36 to 40

☐ 41 to 45

☐ 46 to 50

☐ 51 or older

23. What is your gender?

☐ Male

☐ Female

☐ Other

THANK YOU FOR COMPLETING THIS SURVEY! Please click SUBMIT to record your response.
